# Noninvasive prenatal diagnosis of fetal aneuploidy by circulating fetal nucleated red blood cells and extravillous trophoblasts using silicon-based nanostructured microfluidics

**DOI:** 10.1186/s13039-017-0343-3

**Published:** 2017-12-02

**Authors:** Chung-Er Huang, Gwo-Chin Ma, Hei-Jen Jou, Wen-Hsiang Lin, Dong-Jay Lee, Yi-Shing Lin, Norman A. Ginsberg, Hsin-Fu Chen, Frank Mau-Chung Chang, Ming Chen

**Affiliations:** 1 International College of Semiconductor Technology, National Chiao-Tung University, Hsinchu, Taiwan; 2 Cytoaurora Biotechnologies, Inc. Hsinchu Science Park, Hsinchu, Taiwan; 3 Department of Genomic Medicine and Center for Medical Genetics, Changhua Christian Hospital, Changhua, Taiwan; 4 Department of Genomic Science and Technology, Changhua Christian Hospital Healthcare System, Changhua, Taiwan; 5 Institute of Biochemistry, Microbiology and Immunology, Chung-Shan Medical University, Taichung, Taiwan; 6 Department of Medical Laboratory Science and Biotechnology, Central Taiwan University of Science and Technology, Taichung, Taiwan; 7 Department of Obstetrics and Gynecology, Taiwan Adventist Hospital, Taipei, Taiwan; 8 Department of Obstetrics and Gynecology, College of Medicine, National Taiwan University, Taipei, Taiwan; 9 Welgene Biotechnology Company, Nangang Business Park, Taipei, Taiwan; 10 Department of Obstetrics and Gynecology, Feinberg School of Medicine, Northwestern University Medical Center, Chicago, IL USA; 11 Graduate Institute of Medical Genomics and Proteomics, College of Medicine, National Taiwan University, Taipei, Taiwan; 12 Department of Electrical Engineering, University of California Los Angeles, Los Angeles, CA USA; 13 National Chiao-Tung University, Hsinchu, Taiwan; 14 Department of Obstetrics and Gynecology, Changhua Christian Hospital, Changhua, Taiwan; 15 Department of Medical Genetics, National Taiwan University Hospital, Taipei, Taiwan; 16 Department of Life Science, Tunghai University, Taichung, Taiwan

**Keywords:** cbNIPD, Aneuploidy, fnRBC, EVT, NIPT, Fish, aCGH, NGS

## Abstract

**Background:**

Noninvasive prenatal testing (NIPT) based on cell-free DNA in maternal circulation has been accepted worldwide by the clinical community since 2011 but limitations, such as maternal malignancy and fetoplacental mosaicism, preclude its full replacement of invasive prenatal diagnosis. We present a novel silicon-based nanostructured microfluidics platform named as “Cell Reveal™” to demonstrate the feasibility of capturing circulating fetal nucleated red blood cells (fnRBC) and extravillous cytotrophoblasts (EVT) for cell-based noninvasive prenatal diagnosis (cbNIPD).

**Methods:**

The “Cell Reveal™” system is a silicon-based, nanostructured microfluidics using immunoaffinity to capture the trophoblasts and the nucleated RBC (nRBC) with specific antibodies. The automated computer analysis software was used to identify the targeted cells through additional immunostaining of the corresponding antigens. The identified cells were retrieved for whole genome amplification for subsequent investigations by micromanipulation in one microchip, and left in situ for subsequent fluorescence in situ hybridization (FISH) in another microchip. When validation, bloods from pregnant women (*n* = 24) at gestational age 11–13^+6^ weeks were enrolled. When verification, bloods from pregnant women (*n* = 5) receiving chorionic villus sampling or amniocentesis at gestation age 11^+4^–21 weeks with an aneuploid or euploid fetus were enrolled, followed by genetic analyses using FISH, short tandem repeat (STR) analyses, array comparative genomic hybridization, and next generation sequencing, in which the laboratory is blind to the fetal genetic complement.

**Results:**

The numbers of captured targeted cells were 1–44 nRBC/2 ml and 1–32 EVT/2 ml in the validation group. The genetic investigations performed in the verification group confirmed the captured cells to be fetal origin. In every 8 ml of the maternal blood being blindly tested, both fnRBC and EVT were always captured. The numbers of captured fetal cells were 14–22 fnRBC/4 ml and 1–44 EVT/4 ml of maternal blood.

**Conclusions:**

This report is one of the first few to verify the capture of fnRBC in addition to EVT. The scalability of our automated system made us one step closer toward the goal of in vitro diagnostics.

**Electronic supplementary material:**

The online version of this article (10.1186/s13039-017-0343-3) contains supplementary material, which is available to authorized users.

## Background

Noninvasive prenatal testing (NIPT) that uses cell-free DNA (cfDNA) in maternal circulation for fetal aneuploidy detection had already achieved widespread recognition and adoption by the clinician community worldwide since 2011 [1, 2]. On the other hand, the progress of cell-based noninvasive prenatal diagnosis (cbNIPD) is relatively not so promising or stagnant until very recently [3–8]. Scarcity of fetal cells in the maternal circulation poses a great hurdle to the progress of cbNIPD when compared with much more robust cfDNA-based NIPT. The cfDNA-based testing was conducted through the robust maximal parallel sequencing methods by utility of the high-sensitive, high-throughput, rapid-evolving platforms called next generation sequencing (NGS) technologies which can discriminate the trivial differences between the maternal blood who carry the euploid fetuses and those who carry the aneuploidy fetuses. The NIPT was successfully validated for common fetal chromosomal numerical disorders such as trisomy 13, 18, and 21 [1]. Recently some service providers claimed the repertoire of NIPT can be expanded to all autosomes, and even microdeletion syndromes [9], which is controversial. Most published statements, consensus, or recommendation from the professional societies now consider using NIPT to detect fetal microdeletion syndromes is not recommended [2, 10]. However, cfDNA-based screening heavily relied upon bioinformatics protected by intellectual property which is less easily accessible and thus mainly dominated by the commercial service providers, and had revolutionarily changed the landscape of prenatal diagnosis [2, 11]. Meanwhile, cfDNA-based tests need innovative algorithms to analyze the NGS data, and it is now well known that origins of the cfDNA, in addition to those from maternal, are from the placenta (trophoblasts) instead of from the fetus proper, indicating that fetoplacental mosaicism (namely, the chromosome complements of the fetus and the placenta are different), is an unarguable source of false-negatives and false-positives with the current NIPT [12, 13].

Since 2014, we have developed our in-house patent protected algorithms for cfDNA NIPT (called GWNS™) and the resolution, in some cases, can be even enhanced to a 3.21 Mb microduplication by simply using 20 M reads shallow-sequencing with 12.5% of fetal DNA fraction [14–16]. However, we also noticed the problem of fetoplacental mosaicism [12, 15, 17] and thus re-focused back our effort to cbNIPD since 2015. We believed with the utility of cbNIPD the issue of fetoplacental mosaicism in noninvasive prenatal diagnosis can be better tackled, if we can capture both cells from the fetal and placental origins, namely, the fetal nucleated red blood cells (fnRBC) and the extravillous cytotrophoblasts (EVT).

**Fig. 1:**
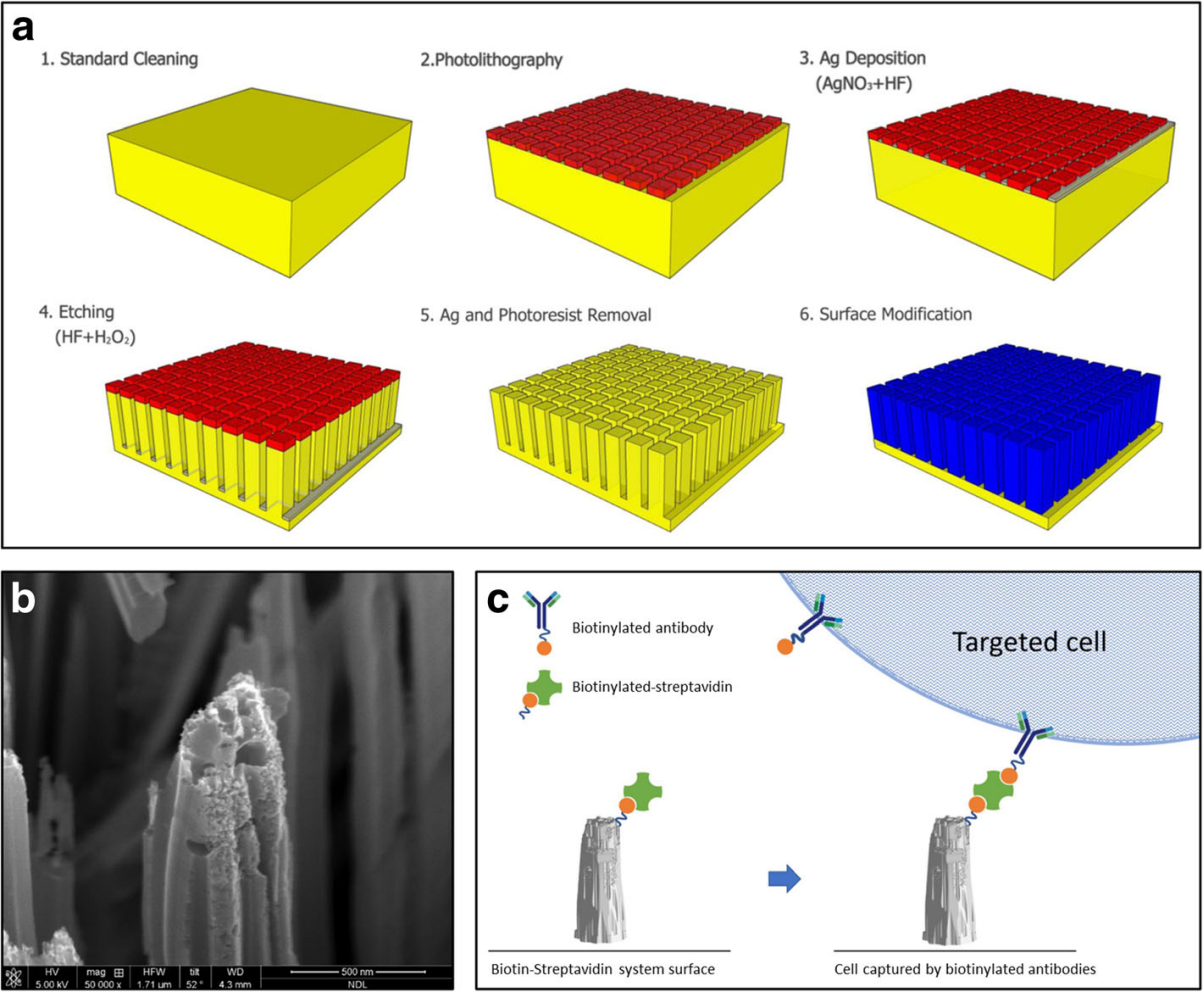
**a** Flowchart of the PicoBioChip manufacture: 1. standard cleaning, 2. photolithography, 3. Ag deposition, 4. etching, 5. Ag and photoresist removal, and 6.surface modification. **b** The porous morphology on the PicoBioChip with a “nano-on-nano” structure. **c** Conceptual illustration of how an PicoBioChip can be employed to achieve significantly enhanced capture of targeted cell

The major difficulty of cbNIPD is the extreme scarcity of fetal cells. It is estimated, by the best recent reports, there were only 1–45 cells per 30 ml maternal blood. And most of the previous efforts captured EVT only, instead of fnRBC, which can truly represent the fetal genome [5]. How to find a feasible method to capture and enrich the fetal cells from the maternal blood become the major challenges of cbNIPD [5, 8]. However, the same technology, if being developed, can be used to capture not only the circulating fetal cells (CFC) but also other cells, including the circulating tumor cells (CTC), and the technologies devised for CTC are numerous: including PCR-based, flow cytometry, laser scanning cytometry, FDA-cleared Cell Search (Veridex, New Jersey, USA), EPISPOT assay [18], and microchip (microfluidics/lab-on-a-chip)-based technologies [19]. Among microchip technologies, the methods had been used to isolate single cells included immune-affinity, immune-magnetic, and size-based methodologies [20]. We selected microchip-based method by using immune-affinity approach which utilizes a microfluidic device (called PicoBioChip, the manufacturing flowchart of the chip was shown in Fig. 1) coated with antibodies which can capture the corresponding antigens on the targeted cells, due to the strength of the semiconductor industry in Taiwan, to devise our novel automated platform Cell Reveal™ to explore the feasibility of cbNIPD to capture CFC (including fnRBC and EVT), firstly by using a group of pregnant women at GA 11–13^+6^ weeks to demonstrate the capability of capturing nucleated RBC (nRBC) and EVT, followed by another group of pregnant woman to verify the captured cells are indeed fetal origin by subsequent genetic investigations, such as fluorescence in situ hybridization (FISH), array comparative genomic hybridization (aCGH), and NGS. The results of cbNIPD are compared with paralleled cfDNA NIPT, and confirmed with invasive procedures using karyotyping, aCGH, and if necessary, NGS. The aim of this small pilot series of proof-of-principle study is to demonstrate the feasibility of our platform with a performance comparable or even superior to the capture efficiencies of other designs previously reported in the literature (which ranged from <1 to 3–5 fetal cells per ml of maternal blood) [4, 6, 8]. Meanwhile, the need of manual handling is reduced to a minimum in our automated system.

## Results

### Circulating fnRBC and EVT captured by PicoBioChip

**Fig. 2:**
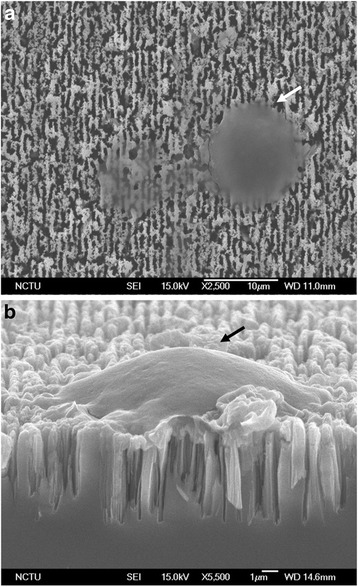
Scanning electron microscope (SEM) micrographs of PicoBioChip: **a** top view and **b** lateral view. Arrow, one captured fetal nucleated red blood cells (fnRBC) or extravillous cytotrophoblasts (EVT)

**Fig. 3:**
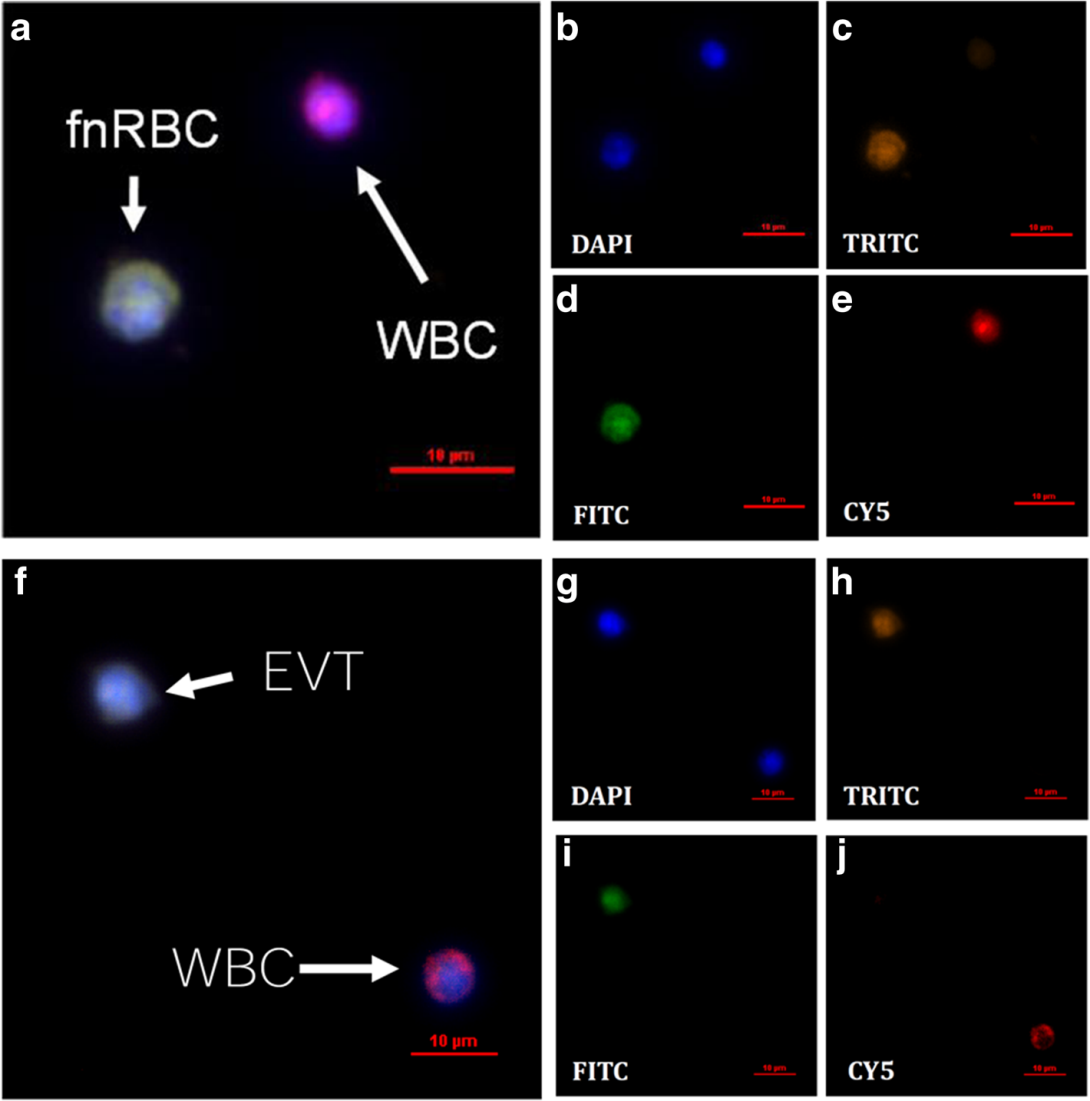
Discrimination of **a-e** fnRBC and **f-j** EVT from maternal white blood cells (WBC) by fluorescence microscope. The fnRBC and EVT can be recognized by different antibodies labeled with TRITC (GPA and HLA-G) or FITC (CD71 and CK7). The maternal leukocytes can be recognized by antibody labeled with CY5 (CD45)

The scanning electron microscope (SEM) micrograph of the PicoBioChip is shown in Fig. 2a. The micrograph demonstrated that the PicoBioChip surface morphology is composed of patterned nanostructure with the same dimension and space, so that circulating cells could be captured by the chip surface (Fig. 2b). The processes of cells capture were automatically performed on a Cell Reveal™ system. By using a fluorescence microscope, the nRBC and EVT can be unequivocally distinguished from the background packed with the WBC from the maternal origin (Fig. 3).

### Capture efficiency

In every 4 ml of the maternal blood used for validation, circulating nRBC and EVT were always captured in all the 24 pregnant women with capture efficiencies as 1–44 nRBC/2 ml and 1-32EVT/ 2 ml (Additional file 1: Table S1). In every 8 ml of the maternal blood used for verification, circulating fnRBC (please refer to the below sections that the nRBCs we captured are indeed fetal origin) and EVT were always captured for all five pregnant women examined (Table 1). A total of 150 fetal cells (fnRBC + EVT) were successfully captured. The numbers of captured fetal cells were: 14–22 cells per 4 ml of maternal blood for fnRBC and 1–44 cells per 4 ml of maternal blood for EVT. The overall capture efficiency of the novel system is estimated as 2.38–7.25 fetal cells (fnRBC + EVT) per ml of maternal blood per individual (Table 2).

**Table 1:** The capture efficiency and related parameters of cell-based prenatal diagnosis by Cell Reveal™ platform with PicoBioChips for 5 pregnant women with an aneuploid or euploid fetus in the verification group

Case no.	Maternal age	Gestational age	Fetal karyotype	Type of fetal cell captured	Captured efficiency^a^ Average (2 ml/2 ml)	FISH^b^	WGA^c^	cfDNA testing^d^
1	36	11^+6e^	47,XX,+13	fnRBC EVT	11 (10/12) 1 (1/1)	10 1	11 (+) 1 (−)	High risk for T13: GWNS: *p* < 0.001 Z score: Z = 8.74
2	34	18^+6^	47,XX,+18	fnRBC EVT	7 (3/11) 22 (11/33)	3 11	11 (+) 15 (+)	High risk for T18: GWNS: *p* = 0.003 Z score: Z = 4.29
3	37	21	47,XX,+21	fnRBC EVT	11 (2/20) 3.5 (3/4)	2 3	15 (+) 4 (+)	High risk for T21: GWNS: p = 0.003 Z score: Z = 3.91
4	30	13^+3^	46,XY	fnRBC EVT	9 (8/10) 1 (1/1)	8 1	6 (+) 2 (−)	Low risk for T13, 18, 21
5	34	11^+4^	46,XX	fnRBC EVT	9 (8/10) 0.5 (0/1)	8 NP	9 (+) 1 (−)	Low risk for T13, 18, 21

*FISH* fluorescence in situ hybridization, *fnRBC* fetal nucleated red blood cells, *GWNS* genome wide normalized score, *NP* Not be performed, *WGA* whole genome amplification

^a^Number of cell captured per 2 ml of maternal blood per PicoBioChip: mean of (chip1/chip2)

^b^Number of cells analyzed

^c^Number of cells pooled for DNA amplification. “+” and “-” indicated the successful amplification and unsuccessful amplification, respectively

^d^Cut-off values of high risk: *p* < 0.05 by GWNS algorithm and z < −3 or >3 by Z score algorithm [14]

^e^11^+6^ denotes 11 weeks and 6 days. cfDNA: cell-free DNA; EVT: extravillous cytotrophoblasts

**Table 2:** The characteristics of the 11 short tandem repeat (STR) loci and one gender-specific locus examined in this study. Primers are labeled with WellRED dye (Beckman Coulter, California, USA)

Locus	Chromosome location	Primer label	Repeat unit length
STR			
D3S1358	3p21.31	D4	4
TH01	11p15.5	D2	4
D13S317	13q31.1	D3	4
D8S1179	8q24.13	D4	4
D7S820	7q11.21–22	D3	4
TPOX	2p25.3	D4	4
D16S539	16q24.1	D3	4
D18S51	18q21.3	D2	4
CSF1PO	5q33.1	D4	4
Penta D	21q22.3	D4	5
Penta E	15q26.2	D3	5
Gender-specific			
AMEL	X and Y	D3	–

### Fluorescence in situ hybridization (FISH)

**Fig. 4:**
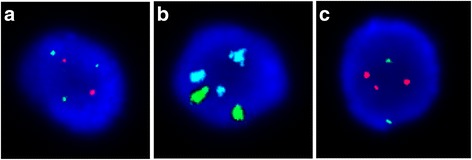
Fluorescent in situ hybridization (FISH) for the captured fnRBC from 3 pregnant women with an aneuploid fetus of **a** trisomy 13, **b** trisomy 18, and **c** trisomy 21. In **a** and **c**, chromosome 13 was identified by a panel of probes (RB1, D13S1195, D13S1155, D13S915) in green and chromosome 21 was identified by a panel of probes (D21S270, D21S1867, D21S337, D21S1425, D21S1444, D21S341) in orange. In **b**, chromosome 18 was identified by a probe (D18Z1) in aqua and chromosome X was identified by a probe (DXZ1) in green

Interphase FISH for the captured fetal cells from the blood of pregnant women with a fetus of trisomy 13, trisomy 18, or trisomy 21 revealed correct diagnoses in all cases. The number of fnRBC and EVT examined ranged from one to ten for each case (Table 1). FISH for the trisomy 13 revealed nuc ish(RB1/D13S1195/D13S1155/D13S915x3, D21S270/D21S1867/D21S337/D21S1425/D21S1444/D21S341x2), for the trisomy 18 revealed nuc ish(D18Z1x3,DXZ1x2), and for the trisomy 21 revealed nuc ish(RB1/D13S1195/D13S1155/D13S915x2,D21S270/D21S1867/D21S337/D21S1425/D21S1444/D21S341x3) (Fig. 4).

### Whole genome amplification (WGA)

All pooled captured cells underwent WGA successfully except those the total numbers of cells were too few (namely, less than 4 cells) to reach the amplified threshold for subsequent molecular genetic analyses by short tandem repeat (STR) analysis, aCGH, and NGS. Overall, fnRBC WGA from all the five cases and EVT WGA from two cases were obtained (Table 1). The WGA products were 50 μl in total with a concentration ranged from 290 to 844 ng/μl.

### Short tandem repeat (STR) analysis

STR analyses were performed for the WGA DNA from captured fetal cells and maternal leukocytes as well as the DNA from the abortus tissue (if available). The results demonstrated the captured fnRBC and/or EVT are indeed fetal origin in all the five cases examined. For each case, there are 4–8 informative STR makers containing non-maternal alleles that are feasible to distinguish the fetal cells from the maternal cells (Table 3).

**Table 3:** Summary of the STR results for the captured fetal cells (fnRBC and/or EVT) from the 5 pregnant women. For each case, at least 4 informative STR loci are feasible to distinguish the fetal cells from the maternal cells (the non-maternal alleles are marked in bold)

Locus	Case 1 (Trisomy 13)		Case 2 (Trisomy 18)				Case 3 (Trisomy 21)			Case 4 (Disomy: 46,XY)		Case 5 (Disomy: 46,XX)	
	Maternal leukocyte	fnRBC	Maternal leukocyte	fnRBC	EVT	Abortus tissue	Maternal leukocyte	fnRBC	EVT	Maternal leukocyte	fnRBC	Maternal leukocyte	fnRBC
D3S1358	133, 137	**129**, 137	129, 137	129, 137	129, 137	129, 137	129, 137	129, 137	129, 137	129, 133	133, **137**	129, 133	129, 133
TH01	171, 179	171, **183**	167, 171	167, **179**	167, **179**	167, **179**	171, 179	171, 179	171, 179	179	179	179	**167**, 179
D13S317	182, 190	182, **198**	197	**181**, 197	**181**, 197	**181**, 197	181, 197	181, 197	181, 197	182, 194	**186**, 194	182, 186	182, 186
D8S1179	222, 234	**218**, 222	238	**218**, 238	**218**, 238	**218**, 238	230, 234	230	230	218, 230	218, 230	231, 239	231
D7S820	234, 238	**230**, 238	234	234, **238**	234, **238**	234, **238**	226, 238	226, 238	226, 238	242, 246	**234**, 242	231, 243	231, **235**
TPOX	272, 276	272	272	272, **284**	272, **284**	272, **284**	272	272, **276**	272, **276**	272, 284	272	272, 284	284
D16S539	285	285	285, 297	**289**, 297	**289**, 297	**289**, 297	284	284	284	289, 297	**293**, 297	285, 301	**297**, 301
D18S51	307, 311	311	303, 315	303, 315	303, 315	303, 315	307, 319	307, **315**	307, **315**	307	307	315, 337	**303**, 315
CSF1PO	315	315, **320**	344	**340**, 344	**340**, 344	**340**, 344	336, 344	336, 344	336, 344	344	**332**, 344	341	341, **349**
Penta D	404, 419	404, 419	405, 414	405, 414	405, 414	405, 414	400, 424	424, **433**	424, **433**	419, 433	419, 433	404, 414	404, **433**
Penta E	418, 451	418	429	**424**, 429	**424**, 429	**424**, 429	414, 419	419, **450**	419, **450**	445, 450	**424**, 450	414, 435	435, **451**
AMEL	105	105	105	105	105	105	105	105	105	105	105, **111**	105	105

### Array comparative genomic hybridization (aCGH) and next generation sequencing (NGS)

**Fig. 5:**
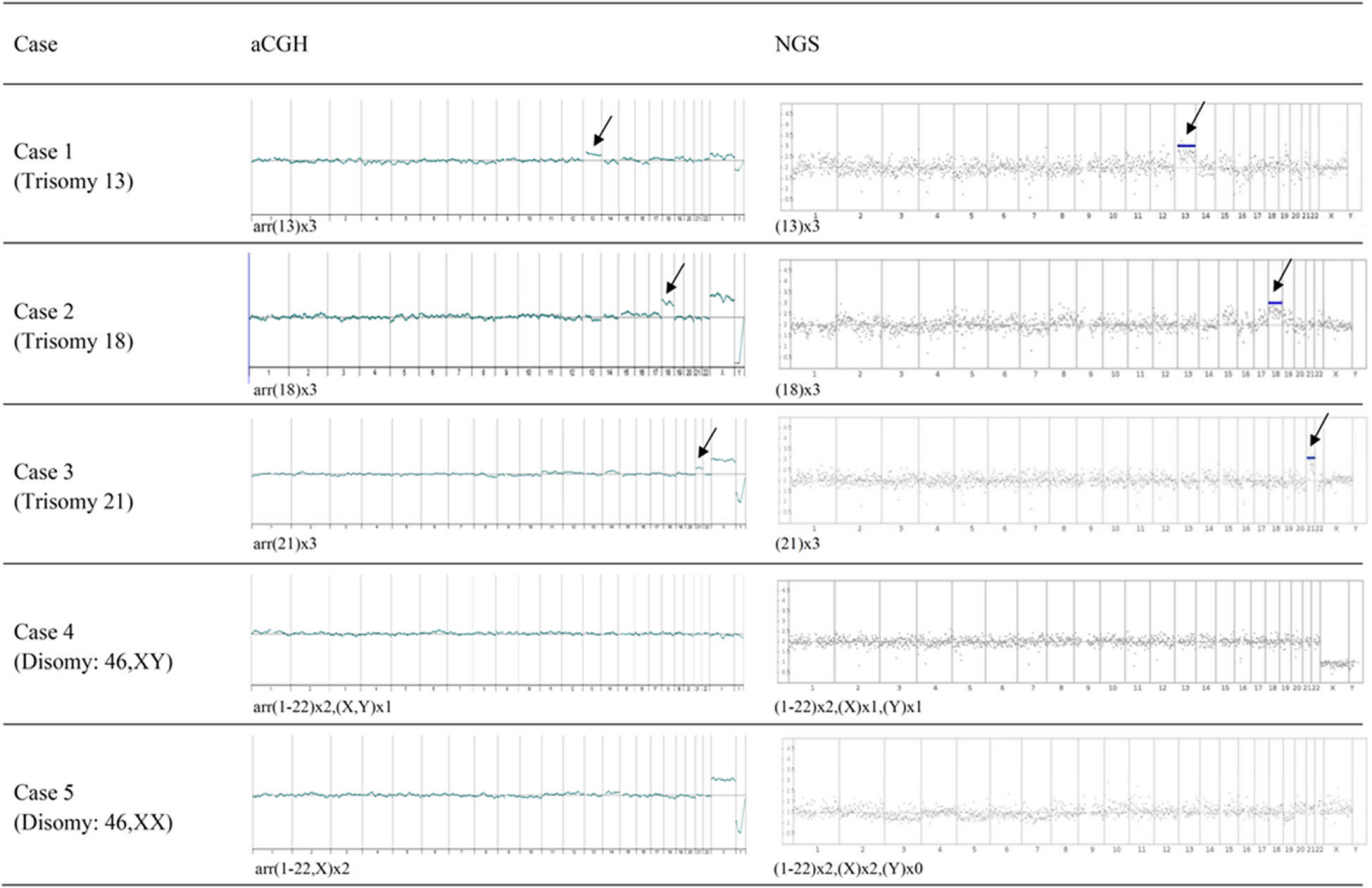
Array comparative genomic hybridization (aCGH) and next generation sequencing (NGS) for the same whole genome amplification (WGA) products of captured fnRBC from 5 pregnant women. WGA product from Promega male DNA (Promega, Wisconsin, USA) was used as reference. The aCGH were performed with GenetiSure Pre-Screen Array Kit 8x60K (Agilent Technologies, CA, USA) on a G4900DA SureScan microarray scanner (Agilent Technologies). NGS was performed using Ion PGM Hi-Q Sequencing Kit with Ion 316 chip (Thermo Fisher Scientific, California, USA) on the Ion Torrent PGM Instrument (Thermo Fisher Scientific) platform. Aneuploidy chromosomes are indicated by arrows. Aneuploidy chromosomes are indicated by arrows. The results of aCGH are comparable with that of NGS

Both of aCGH and NGS analyses were performed for the captured fnRBC from the five cases and all the cases were correctly diagnosed. The results of aCGH are comparable with that of NGS (Fig. 5), and are consistent with the karyotyping results.

## Discussion

The quest to search for a true noninvasive prenatal diagnosis had been the Holy Grail of prenatal diagnosis since 1969 [5]. The major hurdle is the scarcity of fetal cells in maternal circulation, and therefore contributed to the soaring cost of the technologies involved in the enrichment and isolation. Most previous reports found that the majority of nucleated red blood cells isolated from the maternal blood are actually maternal origin instead of fetal [5] whereas a recent published study did isolate fnRBC, confirmed by using chromosome Y-specific FISH [21]. Another group explored the monoclonal antibodies specific for fnRBC in addition to those specific for nucleated RBCs (including both fetal nucleated RBC and nucleated RBC from adult bone marrow origin) such as CD36, CD71, glycophorin-A, antigen-i, and galactose [22]. Unfortunately, it is not commercially available for such monoclonal antibodies claimed to be fetal specific. It is evident that our Cell Reveal™ system with PicoBioChip can capture both the fnRBC and EVT with capture efficiencies superior to or at least comparable to the previous reports (19–58 cells/8 ml v.s. 1–45 cells/30 ml maternal blood), especially all the NRBCs being captured in the verification group by our system are fnRBC. Therefore the feasibility of our platform to tackle the primary hurdle, the scarcity of fetal cells and the difficulty to successfully enrich and capture the very few fetal cells from a limited amount of maternal blood (i.e. 8 ml), is demonstrated. The importance to capture not only the trophoblasts (which is considered placental origin), but also the fnRBC is that the fetoplacental mosaicism can be overcome, otherwise cbNIPD adds not much additional value when being compared with the extremely successful cfDNA-based NIPT, since only fnRBC can genuinely represent the fetal origin instead of placenta origin, which NIPT has already managed [1, 23]. The nucleated red blood cells isolated through our platform are confirmed to be fetal origin by the subsequent analyses including STR analysis, FISH, aCGH, and NGS. Meanwhile, cbNIPD may clearly delineate which co-twin is affected (or much more uncommon, both co-twins are affected) by aneuploidy when the result of cfDNA-based NIPT showed high risk for aneuploidy in twin pregnancies.

In this proof-of-principle pilot study, we only demonstrated the feasibility of our Cell Reveal™ platform to detect fetal aneuploidy by using common trisomies (trisomy 13, 18, and 21). We admit it will be more persuasive if we included some genuine fetoplacental mosaicism cases as some of the cases we published before, and this will be included in our future studies [15, 17, 24]. Some researchers proposed that cbNIPD on EVT can be used to detect de novo copy number variations which can only be reliably diagnosed by microarray, since at the moment the claims by some commercial service providers to expand the repertoire of cfDNA-based NIPT to include microdeletions/microduplications are not widely endorsed by the academic community [5, 10]. However, the errors that may be introduced by the indispensable WGA procedure had also been noticed recently from the experience obtained from the studies regarding preimplantation genetic screening. More and more reports were published since 2015 that euploid babies were born after transferring the aneuploid embryos into the womb [25] and the consistency of PGS across the laboratories adopting different genotyping technologies is questioned [26–29]. Thus, we considered aCGH and NGS can be used but should be interpreted with caution because WGA is the necessary step before using these technologies. A better capture efficiency to capture more cells and group them together for the subsequent analyses will be very helpful to minimize the errors introduced by WGA. In our laboratory, the previous experience on PGD/PGS made us only did WGA on at least four cells, therefore we did not do WGA on the EVT captured on Case no. 1, 4, 5 whereas we did WGA on the nRBC we captured on all the five cases in the verification group (Table 1). Remarkably, most previous reports in the literature regarding cbNIPD using different methods such as immunoaffinity by magnetic enrichment [8] or NanoVelcro microchip [30], or isolation by size of tumor/trophoblast cells (ISET) [31] only successfully isolated trophoblasts instead of fnRBC. However, it is remarkable that ISET was reported to be able to isolate living cells [32].

On the other hand, it is now better known the trend of the variation of the fetal DNA fractions during the whole gestation, as well as the possible confounding factors, since the fetal fraction is one of the major factors affecting the accuracy of NIPT [10, 33]. Needless to say, such similar large-scale studies are mandatory for cbNIPD in order to better understand the variation as well as the confounding factors affecting the numbers of both types of the fetal cells (fnRBC and EVT), to facilitate its wider acceptance of clinical utility. The future studies will be ideal if including some fetoplacental mosaicism cases as well as twin pregnancies with one or two aneuploidy cases to better demonstrate its feasibility to supplement the current cfDNA-based NIPT. Lastly, another critical issue affecting the uptake of cbNIPD by the clinical community in the future is the cost. A detailed cost-effective analysis is needed in the future for cbNIPD as it has been done in NIPT for fetal aneuploidy [34]. Nevertheless, this platform has the potential to be used for capturing the circulating tumor cells (CTC) as well [32]. It is arguably that the nRBC captured in the validation group (*n* = 24) may both include the fetal and the maternal nRBC (these cells are released from the adult bone marrow) since we did not use the fetal specific monoclonal antibodies such as those recognize the Epsilon hemoglobin [35]. However, the subsequent genetic investigations we performed (including FISH, aCGH, STR, and NGS) had verified the captured cells in the verification group (*n* = 5) are indeed fetal origin.

To the best of our knowledge, this report is one of the very few studies on the successful use of circulating fetal cells for noninvasive prenatal diagnosis. The strength of our study is that all the processes of cell capture are automatic which can be performed on a single individual case and completed within 15 h (Additional file 2: Table S2). The captured cells are available for a variety of genetic testing, such as FISH, aCGH and NGS. Overall, the turnaround time of the cbNIPD is less than 2 weeks, similar to that of NIPT as performed in our laboratory.

## Conclusion

We demonstrated our silicon-based nanostructured microfluidics “The Cell Reveal™ system” can capture both the fnRBC and EVT. The scalability is greatly enhanced with the automation of the whole system, which may render cbNIPD from mainly a laboratory-developed-test (LDT) conducted only in a limited number of core laboratories, into an in-vitro-diagnostics (IVD) that can be applied in many research and clinical sites.

## Methods

### Samples

During 2016–2017, 24 women who carried the singleton pregnancy and received the first trimester serum screening for Down syndrome at GA 11–13 + 6 weeks or who decided to receive NIPT, was asked to donate blood sample to be used for validation. For each individual, 4 ml additional blood was stored in the BD vacutainer® with ACD solution A (Becton, Dickinson and Company, New Jersey, USA) for cbNIPD. When verification, at 2017 another five pregnant women carrying the singleton pregnancy at first or second trimester who decided to receive invasive procedures (chorionic villus sampling or amniocentesis) were recruited as a research basis to receive paralleled cfDNA testing (i.e. NIPT) and cbNIPD after informed consents (with the approved protocol CCH-IRB-141219) were signed. For each individual, approximately 20 ml of venous blood were collected. The blood was taken and stored in the Streck Cell-Free DNA BCT® (Streck, Nebraska, USA) for NIPT (12 ml) and in the BD vacutainer® with ACD solution A (Becton, Dickinson and Company, New Jersey, USA) for cbNIPD (8 ml). A total of 3 pregnant women who had singleton pregnancy affected with fetal aneuploidy were recruited, including trisomy 13 (*n* = 1), trisomy 18 (n = 1), and trisomy 21 (n = 1) fetuses respectively. Meanwhile, two women carrying the euploid fetuses (46,XX, n = 1 and 46,XY, n = 1) were also enrolled. The pregnant women were enrolled at first or second trimester ranged from gestational age 11^+4^ to 21 weeks (Table 1). It is noteworthy that the examiners of the cbNIPD lab have no prior knowledge of the karyotyping results, namely, they were blind to the results to avoid ascertainment bias. The recruitment of patients, collection of samples, and conduct of research projects, were approved by the Ethical Commitees of the medical institutions where the samples were collected (the Taiwan Adventist Hospital, Taipei, Taiwan, and the Changhua Christian Hospital, Changhua, Taiwan).

### PicoBioChip manufacture

The PicoBioChip is a Si nanostructure with a porous morphology that is fabricated using the metal-assisted chemical etching (MACE) technology. The fabrication sequence is described as followed: the starting materials are p-type (100) silicon wafers which followed standard cleaning procedures to remove environmental contaminants. The pattern of the PicoBioChip is defined by standard photolithographic techniques. The Ag film is deposited onto the silicon wafer in a HF/AgNO_3_ mixture solution, and the wafers are etched in a HF/H_2_O_2_mixture solution. Then, after the etching step and the Ag film removal, a Si nanostructure with a porous morphology is formed that is a “nano-on-nano” structure. To enhance the capturing effect, the potential targeted cells are pre-labeled with biotinylated antibodies and the PicoBioChip surface is made from a streptavidin material which has a specific binding interaction with biotin. The streptavidin-biotin is the strongest non-covalent biological interaction currently known. Via streptavidin-biotin interaction, biotinylated antibodies can be conjugated, enabling a high efficiency for targeted cells capture. The manufacturing flowchart, nano-on-nano structure and capture conception of the PicoBioChip are shown in Fig. 1.

### Circulating fetal cells captured by cell reveal™ system with PicoBioChip

The whole blood sample (8 ml) is flown through the automated Cell Reveal™ system and then CFC are captured by PicoBioChips. For each run of test, four PicoBioChips were used: two for fnRBC capture and two for EVT capture. The antibodies used for primary capture of circulating fetal cells are CD71^+^ for fnRBC and EpCAM^+^ for EVT. PicoBioChips are examined using a fluorescence microscope equipped with a built-in automatic inspection and image analysis system, called the Cell Analysis Tool (CytoAurora CAT™), to filter out images of maternal white blood cells (WBC) for further analyses. The fnRBC and EVT can therefore be targeted, identified and enumerated. Image analyses with the count-in/filter-out criteria for different cell types are CD71(+)/GPA^+^(glycophorin-A)/CD45^−^/DAPI^+^ for fnRBC and CK7^+^(Cytokeratin-7)/HLA-G(+)/CD45^−^/DAPI^+^ for EVT, according to literatures and our in-house optimization [6, 21, 22, 35–40]. Namely, we first used one antibody to capture fnRBC and EVT separately, and then using other antibodies to stain the captured cells. Hence, the fnRBC were primarily captured by CD71 and then stained with CD71 and GPA, whereas the EVT were primarily captured by EpCAM and then stained with CK7 and HLA-G. Namely, we utilized dual antibodies (CD71 and GPA) to delineate the fnRBC and triple antibodies (EpCAM, CK7, HLA-G) to delineate EVT. It is noteworthy that in the validation group (*n* = 24) to validate the capture efficiency, only 4 ml maternal blood was used, in which 2 ml was for fnRBC (or more strictly, nRBC) and 2 ml was for EVT in each case. Only the five pregnant women enrolled for verification had 8 ml maternal blood to be withdrawn and used for cbNIPD.

### Fluorescence in situ hybridization (FISH)

FISH was performed directly on one PicoBioChip capturing for fnRBC and one chip for EVT. Prior to hybridization, the formaldehyde on PicoBioChips were treated by 10 mM sodium citrate at 90 °C for 20 min, followed by being immersed in 0.1% Triton-X at room temperature for 10 min, then followed by serial washes of 0.2 N HCl at 25 °C for 20 min, purified water (double distilled) at 25 °C for 3 min and 2X SSC at 25 °C for 3 min, and an immersion of Vysis pretreatment solution (1 N NaSCN) (Abbott, IL, USA) at 25 °C overnight. Then, the PicoBioChips were deposited in purified water at 25 °C for 1 min, 2X SSC at 25 °C for 5 min (repeated two times), pepsin solution (10 μl 10% Pepsin / 40 ml 0.01 N HCl) at 37 °C for 3 min and 2X SSC at 25 °C for 5 min (repeated two times). Finally, the PicoBioChips were immersed in 70% ethanol at 4 °C for 1 min, 85% ethanol at 4 °C for 1 min and 100% ethanol at 4 °C for 1 min, and dried at 50 °C for 5 min. Interphase FISH for chromosome 13, 18 and 21 on captured fnRBC and EVT was then conducted using Aquarius® FAST FISH Prenatal kit (Cytocell, Cambridge, UK). For hybridization experiment, the PicoBioChips were dehydrated in an ethanol series and hybridized overnight in a moist chamber at 37 °C. The chips were washed for 2 min in 0.4X SSC at 70 °C and for 5 min in 4X SSC, 0.1% Tween 20 at room temperature and blocked in 4X SSC, 3% bovine serum albumin (BSA), 0.1% Tween 20 at 37 °C for 30 min. The hybridization signal was detected with Nikon-Ni-E microscope system (Nikon, Tokyo, Japan). Chromosomes were counterstained with 0.125 μg/ml DAPI in Antifade (Vysis, Illinois, USA). FISH analyses were performed using the Aquarius® FAST FISH Prenatal kit (Cytocell). The chromosome 13 probe for RB1, D13S1195, D13S1155, and D13S915, the chromosome 18 probe for centromere of chromosome 18(D18Z1), the chromosome 21 probe for D21S270, D21S1867, D21S337, D21S1425, D21S1444, and D21S341, and the chromosome X probe for centromere of chromosome X (DXZ1) were labeled with green, aqua, orange, and green fluorophores, respectively.

### Retrieval of captured cells by PicoBioChip

The captured fnRBC and EVT are separately released by with capillary micropipette from PicoBioChips which are destined for DNA analyses. The location of captured cells-on-chip is acquired by the CytoAurora CAT™. Capillary micropipette crashes the chip’s nano structure of the target captured cells. The captured cells on the chip surface are followed by capillary micropipette picking up, which allows captured cells to escape from chip to be released for sequential analyses.

### Whole genome amplification (WGA)

The captured fetal cells retrieved from the same PicoBioChip are pooled. The fnRBC and EVT were subjected separately to WGA, with 1.8 μg/μl BSA serving as the blocking agent to reduce the surface interaction from the silicon debris. WGA was performed using REPLI-g Single Cell Kit (Qiagen, Hilden, Germany) and following the manufacturer’s instructions. Amplified DNA was purified using the QIAamp DNA Blood Mini Kit (Qiagen). The DNA purities and concentrations were examined by Qubit fluorometer (Thermo Fisher Scientific, Delaware, USA) and Nanodrop 2000 spectrophotometer (Thermo Fisher Scientific).

### Short tandem repeat (STR) analysis

STR analysis was performed to confirm that the circulating cells captured and WGA DNA of fnRBC and EVT are indeed from fetuses instead of maternal origin. GenomeLab Human STR Primer Set kit (Beckman Coulter, California, USA) containing 12 primer pairs to amplify 11 STR loci and one gender-specific locus (Table 2) was used to analyze patterns of the STR by capillary electrophoresis according with the supplier’s protocol. PCR products were run on GenomeLab™ GeXP Genetic Analysis System (Beckman Coulter). FRAGMENTS application program (Beckman Coulter) was used for data collection and allele sizing.

### Array comparative genomic hybridization (aCGH)

Approximately 1000 ng of WGA DNA was subjected to aCGH by GenetiSure Pre-Screen Array Kit 8x60K (Agilent Technologies, CA, USA), following the manufacturer’s instructions. The image on a chip was acquired with a G4900DA SureScan microarray scanner (Agilent Technologies, CA, USA) and analyzed with Agilent CytoGenomics software (Agilent Technologies) for chromosome gain or loss. Aberrations were detected by using default setting.

### Next generation sequencing (NGS)

Approximately 1000 ng of WGA DNA was used for library construction using Ion Xpress Plus gDNA Fragment Library Preparation Kit Set (Thermo Fisher Scientific, California, USA) and following the manufacturer’s instructions. The quantity of library was determined using Qubit dsDNA HS assay kits (Thermo Fisher Scientific) with Qubit fluorometer (Thermo Fisher Scientific). The template-positive Ion Sphere Particles were generated using Ion PGM Hi-Q Template Kits (Thermo Fisher Scientific) with the Ion OneTouch 2 Instrument (Thermo Fisher Scientific) and then enriched with the Ion OneTouch ES Instrument (Thermo Fisher Scientific). Sequencing was performed on the Ion Torrent PGM Instrument (Thermo Fisher Scientific) platform using the Ion PGM Hi-Q Sequencing Kit and Ion 316 chip (Thermo Fisher Scientific). Analysis of the WGA product being sequenced was performed by using the cloud-based the Ion Reporter™ Server System (https://ionreporter.thermofisher.com/ir/).

## Additional files

Additional file 1: Table S1. The numbers of nRBC and EVT captured in the 24 validated cases. (DOCX 17 kb)

Additional file 2: Table S2. The timeframe of the fetal cell capture from maternal blood by Cell Reveal^TM^ system. (DOCX 18 kb)

## Abbreviations

aCGH: Array comparative genomic hybridization
BSA: Bovine serum albumin
cbNIPD: Cell-based noninvasive prenatal diagnosis
CFC: Circulating fetal cells
cfDNA: Cell-free DNA
CTC: Circulating tumor cells
EVT: Extravillous cytotrophoblasts
FISH: Fluorescence in situ hybridization
fnRBC: Fetal nRBC
GWNS: Genome wide normalized score
MACE: Metal-assisted chemical etching
NGS: Next generation sequencing
NIPT: Noninvasive prenatal testing
nRBC: Nucleated red blood cells
SEM: Scanning electron microscope
STR: Short tandem repeat
WBC: White blood cells
WGA: Whole genome amplification
