# Supplementary material for: Noninvasive prenatal diagnosis of fetal aneuploidy by circulating fetal nucleated red blood cells and extravillous trophoblasts using silicon-based nanostructured microfluidics
Source: Mol Cytogenet. 2017 Dec 2;10:44. doi: 10.1186/s13039-017-0343-3 (PMC5712079; doi:10.1186/s13039-017-0343-3)
Supplement: Supplementary file 1 — The numbers of nRBC and EVT captured in the 24 validated cases. (DOCX 17 kb) [file 13039_2017_343_MOESM1_ESM.docx]

**Additional file 1: Table S1** The numbers of nucleated red blood cell (nRBC) and extravillous cytotrophoblast (EVT) were captured in 24 validated cases.

| Case no. | Gestational age  (Weeks^+days^) | nRBC | EVT |
| --- | --- | --- | --- |
|  |  |  |  |
| 1 | 12^+2^ | 5 | 1 |
| 2 | 12 | 7 | 1 |
| 3 | 12^+3^ | 17 | 8 |
| 4 | 11^+3^ | 28 | 12 |
| 5 | 12^+1^ | 5 | 3 |
| 6 | 12^+1^ | 3 | 2 |
| 7 | 12^+2^ | 5 | 1 |
| 8 | 12^+4^ | 44 | 32 |
| 9 | 12 | 2 | 23 |
| 10 | 11 | 4 | 6 |
| 11 | 12 | 25 | 3 |
| 12 | 11 | 2 | 1 |
| 13 | 12 | 9 | 9 |
| 14 | 12 | 13 | 4 |
| 15 | 12^+6^ | 5 | 11 |
| 16 | 12^+3^ | 23 | 1 |
| 17 | 12^+5^ | 12 | 2 |
| 18 | 13^+4^ | 1 | 13 |
| 19 | 12^+1^ | 6 | 5 |
| 20 | 12^+2^ | 3 | 1 |
| 21 | 12^+3^ | 3 | 10 |
| 22 | 13^+6^ | 5 | 5 |
| 23 | 13^+2^ | 1 | 19 |
| 24 | 12^+5^ | 4 | 5 |
|  |  |  |  |
