# Supplementary material for: Noninvasive prenatal diagnosis of fetal aneuploidy by circulating fetal nucleated red blood cells and extravillous trophoblasts using silicon-based nanostructured microfluidics
Source: Mol Cytogenet. 2017 Dec 2;10:44. doi: 10.1186/s13039-017-0343-3 (PMC5712079; doi:10.1186/s13039-017-0343-3)
Supplement: Supplementary file 2 — The timeframe of the fetal cell capture from maternal blood by Cell RevealTM system. (DOCX 18 kb) [file 13039_2017_343_MOESM2_ESM.docx]

**Additional file 2: Table S2** The timeframe of the fetal cell capture from maternal blood by Cell Reveal^TM^ system.

| Procedure | | Operating time (minute) |
| --- | --- | --- |
| 1. Peripheral blood mononuclear cells (PBMC) isolation from whole blood | |  |
|  | - 1. PBMC isolation by centrifugation | 50 |
|  | - 1. PBMC wash by wash medium | 10 |
|  | - 1. PBMC capture (antibody incubation) | 45 |
|  | - 1. Centrifugation | 10 |
| 2. Fetal cell capture, staining and retrieval | |  |
|  | 2-1. Leakage test | 30 |
|  | 2-2. Cell capture | 30 |
|  | 2-3. Cell fixation | 40 |
|  | 2-4. Washing | 40 |
|  | 2-5. Triton | 50 |
|  | 2-6. Washing | 20 |
|  | 2-7. Blocking | 30 |
|  | 2-8. Primary antibody incubation | 240 |
|  | 2-9. Washing | 40 |
|  | 2-10. Secondary antibody incubation | 80 |
|  | 2-11. Washing | 40 |
|  | 2-12. DAPI staining | 80 |
|  | 2-13. Captured cell retrieval | 30 |
| Overall | | 865 |
